# Food Adulterations

**Published:** 1887-04

**Authors:** 


### FOOD ADULTERATIONS.

If there ever was a time when the strong arm of the law should be interposed to prevent the pestiferous adulterations of food it is now. Almost daily some new fact is brought to light which shows how general and wide-spread these dishonest practices have become. They not only enter largely into the luxuries of life, but the substantials also. Jellies and glucose, which possess scarcely any life sustaining qualities, find their way into almost everything that they can be made to imitate, whilst a hundred other such like trash are brought to light under chemical analysis. Where this is so freely allowed as it is in New York, the consumer can never know what he is eating or drinking. A sneak thief is lurking in all his provisions. There is scarcely an exception. One can hardly say grace without a mental reservation. The whole infernal tribe of false pretenders should be cleaned out; they are worse than a litter of rats in a grain loft. Bread and water for sixty days would be only too good for them.

